# Evaluating a linear ***k***-mer model for protein-DNA interactions using high-throughput SELEX data

**DOI:** 10.1186/1471-2105-14-S10-S2

**Published:** 2013-08-12

**Authors:** Juhani Kähärä, Harri Lähdesmäki

**Affiliations:** 1 Department of Information and Computer Science, Aalto University School of Science, FI-00076 Aalto, Finland; 2 Turku Centre for Biotechnology, Turku University, Turku, Finland

## Abstract

Transcription factor (TF) binding to DNA can be modeled in a number of different ways. It is highly debated which modeling methods are the best, how the models should be built and what can they be applied to. In this study a linear *k*-mer model proposed for predicting TF specificity in protein binding microarrays (PBM) is applied to a high-throughput SELEX data and the question of how to choose the most informative *k*-mers to the binding model is studied. We implemented the standard cross-validation scheme to reduce the number of *k*-mers in the model and observed that the number of *k*-mers can often be reduced significantly without a great negative effect on prediction accuracy. We also found that the later SELEX enrichment cycles provide a much better discrimination between bound and unbound sequences as model prediction accuracies increased for all proteins together with the cycle number. We compared prediction performance of *k*-mer and position specific weight matrix (PWM) models derived from the same SELEX data. Consistent with previous results on PBM data, performance of the *k*-mer model was on average 9%-units better. For the 15 proteins in the SELEX data set with medium enrichment cycles, classification accuracies were on average 71% and 62% for *k*-mer and PWMs, respectively. Finally, the *k*-mer model trained with SELEX data was evaluated on ChIP-seq data demonstrating substantial improvements for some proteins. For protein GATA1 the model can distinquish between true ChIP-seq peaks and negative peaks. For proteins RFX3 and NFATC1 the performance of the model was no better than chance.

## Introduction

Many proteins bind DNA and do that in a sequence specific way. These DNA-binding proteins include transcription factors (TF), among others, which have an important function in regulating gene expression by affecting transcription and chromatin state. Given the fundamental role of TFs in cellular processes and difficulty in measuring their binding sites, computational analysis of binding sites can provide tremendous help in understanding complex regulatory mechanisms [1]. The DNA preference of DNA-binding proteins can be modelled with different computational methods [2]. All methods require known binding sites or data from biological experiments, such as gene expression profiling, chromatin immunoprecipitation followed by sequencing (ChIP-seq), protein binding microarrays (PBM) or systematic evolution of ligands by exponential enrichment (SELEX) followed by sequencing, to build the models.

The modeling method which has become the major paradigm is the position specific weight matrix (PWM) model [3,4]. Position weight matrices are constructed by using sequences from an experiment or automatically aligning binding sites within longer sequences [5,6]. The number of each base is calculated in each position of the alignment, and then each base is assigned a score based on the counts. This way each position treats the nucleotides independently from the other positions: the score is based only on the frequency of the base in that certain position. Consequently, PWMs have been criticized that they might lose some important dependencies between nearby nucleotides, but PWMs provide a very easy and intuitive modeling framework and, moreover, thousands of different PWMs exist in several databases [7,8].

*k*-mer models offer a different perspective in DNA-protein interactions. In *k*-mer models each *k*-mer, a nucleotide sequence of length *k*, is given a score that describes the binding affinity of the protein towards the *k*-mer. The score can be assigned to each *k*-mer for example by utilizing the distribution of *k*-mers in the binding sites, or solving the score using modeling approaches. *k*-mer models help in capturing depedencies between the nucleotide positions in the binding. A recent comparison study demonstrated that *k*-mer models can provide equal or better performance compared to traditional PWM models, particularly so on high-throughout PBM data [2]. On contrary, performance of *k*-mer models relative to PWM-based methods was found to severely degrade when tested on *in vivo *data.

Here we focus on the *k*-mer model by Annala *et al. *[9] which was ranked the first in the original DREAM5 challenge and was among the two most accurate methods in a comprehensive comparison by Weirauch *et al. *[2]. We apply the *k*-mer model to 15 proteins for which SELEX data is available [10] and, thus, report performance of the *k*-mer model on another high-throughput protocol complementary to PBM data used in [2]. The performance is also compared against PWM models derived from the same data.

To improve robustness and generalization of *k*-mer models we studied different feature selection strategies for choosing the most informative *k*-mers to the model. The first feature selection strategies were to select the most frequent or the most enriched *k*-mers. In addition we implemented the cross-validation scheme for choosing the *k*-mers. Finally, the *k*-mer model trained with SELEX data was evaluated on ChIP-seq data with varying results.

## Materials and methods

### SELEX data

In SELEX protocol [10] the experiment starts with a large pool of all different DNA-sequences of fixed length. The protein of interest is introduced into the pool, and the protein will recognize and bind its target sequences. Then an antibody is added to the solution and the protein-DNA complexes are immunoprecipitated. The bound sequences are then amplified and sequenced. The process is repeated by using the bound sequences as a new initial pool of sequences. The protocol produces massive amount of bound sequences (reads) for different iterations which can be used to construct models for describing DNA-protein interactions as well as to evaluate their predictive performance.

The SELEX data from [10] contained enriched reads from the SELEX process for different enrichment cycles. To account for non-specific carryover of DNA from previous cycles, a specially designed multinomial method was used to construct PWMs from high-throughput SELEX data in [10]. The obtained PWMs were found to be very similar with those obtained by the standard MEME [5], thus providing us a good benchmark. The PWM-models were however constructed using only one cycle, and only that cycle was taken into consideration in the performance comparison between *k*-mer and PWM models. Cycles that were included consisted between 9000 and 175000 reads with average being 62000 reads. These sets of reads were randomly divided to training and test sets with ratio 7:3. The training set was used for training the *k*-mer model, and the test set was used in the performance analysis and comparisons. Random reads (negative set) were generated to match the number of enriched reads for each training and testing set. The choice to use uniformly random sequences instead of picking random genomic locations was justified by the fact that in the original experiment the initial pool of nucleotide sequences included evenly all 14-mers. The model performance and comparison was evaluated by a classification task where the test data set consisting of enriched reads not used in the training and equal amount of random reads.

### Description of the linear ***k***-mer model

The linear *k*-mer model presented by Annala *et al. *[9] assigns an affinity score to each *k*-mer and the total binding affinity to a certain sequence is the sum of the binding affinities of the *k*-mers in that sequence. In the original work this affinity was measured by signal strength of a spot in PBM. The affinity score for the *k*-mers can be solved from the linear model *Ax *= *b *+ *ε*, where *A *is the design matrix, *x *is the affinities (column vector), *b *is signal strength and *E *represents noise. In the design matrix each row represents a probe and columns are the variables, the *k*-mers. The *A_ij _*element of the matrix is one if the *k*-mer corresponding to the *j*th column can be found in the *i*th probe, zero otherwise.

The *k*-mer model is trained by solving the linear equation *Ax *= *b*. In the original application for PBMs the *b *vector resulting from the multiplication consisted of probe intensities. For SELEX experiments no intensity data is measured but instead the *b *vector is a binary vector where one denotes a detected enriched (*i.e*. bound) read and zero denotes a random read (unbound). The training requires both bound and unbound sequence reads in order to produce useful predictive models. In this model training scheme the reads were treated as equally important, which might not be true, as the binding affinity of a protein will vary between individual reads. However, given sufficient sequencing depth, the substantial number of reads could compensate this assumption because strongly bound *k*-mers would occur more frequently in the data.

### The choice of ***k***-mers

A key question with the linear *k*-mer model is which *k*-mers should be included into the model. To begin with, we chose two ways to do this. First way was to choose the most common *k*-mers in the whole data, the second way was to choose the most enriched *k*-mers. The enriched *k*-mers were picked from the *k*-mer table which lists the counts of each *k*-mer in the set of bound reads and random unbound reads and sorts them based on either the difference or fold change of those counts. The higher the difference or fold change, the more enriched the *k*-mer in question is. The standard cross-validation scheme was also implemented. First we started with a predefined number of most frequent *k*-mers, and then we started removing those *k*-mers from the model, whose removal improved the results most or had the smallest negative effect in 10-fold cross-validation classification within the training set, essentially implementing a wrapper type of feature selection technique. The number of *k*-mers in the beginning should not be too high, because cross-validation computation gets heavy easily. Note that, to avoid the selection bias [11], the final prediction performance for the cross-validated model was assessed on the separate testing data used neither during the model training nor during the feature selection.

## Results

The *k*-mer models trained as explained above and the PWM models taken from Jolma et al. [10] were used in classifying the reads in the testing set. Note that the PWM models were derived using a combination of training and testing data, which may slightly positively influence the PWM results. The classification using PWMs was conducted by scanning the reads in the testing set with the given model and the maximum of the scores was assigned to that read. The reads were classified using threshold that was found optimal in the training set. Classification accuracy is estimated as the proportion of reads that are classified correctly. The confidence intervals are normal approximation intervals.

**Figure 1:**
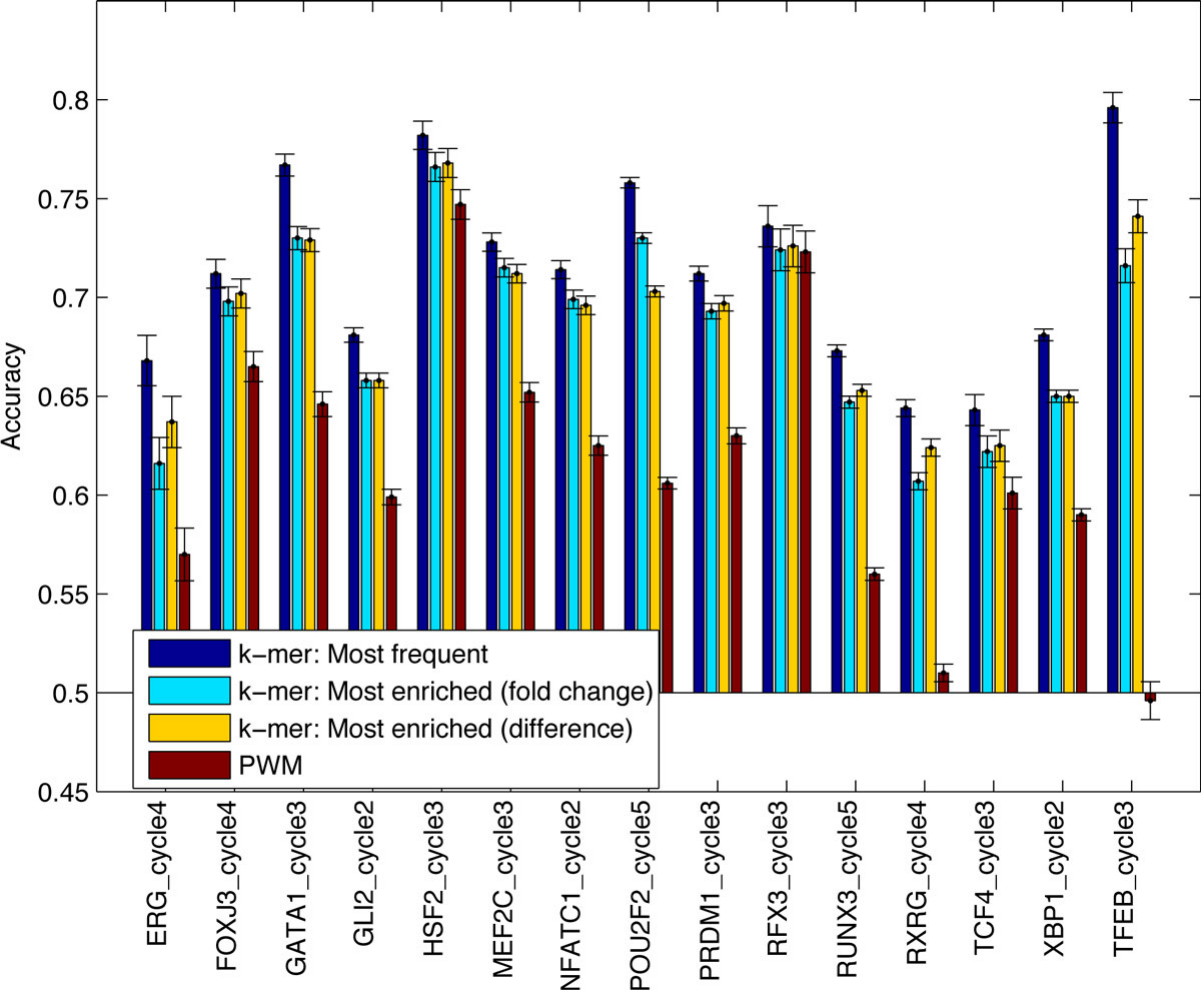
**Classification accuracies of the ***k***-mer model and PWM models**. The test set classification accuracy is plotted for 3 different *k*-mer model approaches and the PWM model. The 95% normal approximation confidence intervals are plotted on top of each bar.

The classification accuracies together with 95% confidence intervals are shown in Figure 1. The *k*- mer model clearly outperforms the PWM models as can be seen from the figure. In the first bars the accuracy of the *k*-mer model is the accuracy using the optimal number of most frequent *k*-mers. The average classification accuracy is 71% for the *k*-mer model and 62% for the PWM models. In the second and third bars the accuracy is obtained with *k*-mer model using the most enriched *k*-mers. Surprisingly *k*-mer model performance is better, when choosing the most frequent *k*-mers instead of the most enriched *k*-mers. This might indicate that assigning high affinity scores to *k*-mers responsible for binding as well as giving negative affinity scores to frequent *k*-mers that are not part of the binding are important.

**Figure 2:**
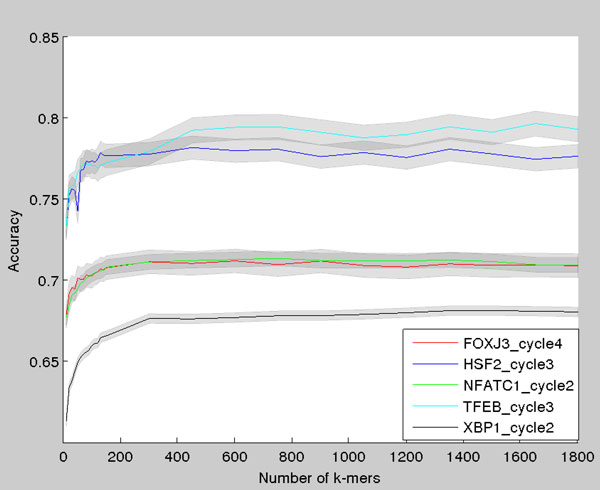
**Classification accuracy as a function of number of ***k***-mers in the model**. The test set classification accuracy plotted as a function of number of *k*-mers in the model. The 95% normal approximation confidence intervals are plotted around each curve as grey area.

The effect of the number of *k*-mers in the model is shown in Figure 2. The average classification accuracy increases sharply when *k*-mers are added to the model, and the accuracy reaches its maximum at about 600 *k*-mers. Using more than 600 *k*-mers has little effect on results. However, for individual proteins the classification accuracy seems to peak at around 600 or 1500 *k*-mers. In later cycles the data can be classified with great accuracy by using only one *k*-mer. For protein XBP1 it suffices to include only *k*-mer ACGT to the model, and the data can be classified with accuracy of 91%. It is worth noting, that the *k*-mer is its reverse complemented and can be therefore detected from both strands.

### Selection of ***k***-mers improves prediction accuracy

**Figure 3:**
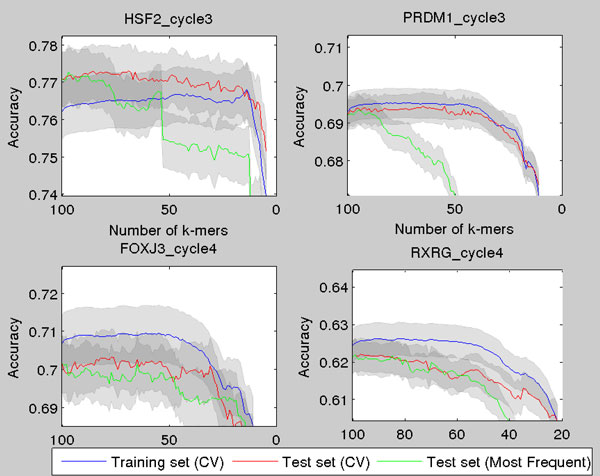
**Illustration of classification accuracy using the CV-scheme for four proteins**. The test set classification accuracy as a function of number of *k*-mers, when the *k*-mers are chosen with the CV-scheme. The CV is started from 100 most frequent *k*-mers. The 95% normal approximation confidence intervals are plotted around the curves.

The cross-validation gives a slight improvement to the results. If we start with for example 200 *k*-mers, we can end up in a set of 100 *k*-mers which is better than taking just the 100 most frequent *k*-mers--the main motivation for feature selection in discriminatory analysis. The change in classification accuracy, when starting with 100 *k*-mers, is shown for four proteins in Figure 3. The blue line represents the accuracy in the 10-fold cross-validation within the training set, and red line is the classification accuracy in the testing set. Green line represents the classification accuracy, when the model is trained using the equal number of *k*-mers that are most frequent in the data. Horizontal axis represents the number of *k*-mers in the model.

The binding prediction results vary between proteins. For example for PRDM1 and HSF2 the cross-validation scheme can choose *k*-mers producing much better classification results. The data can be classified reliably with only seven *k*-mers (HSF2). With FOXJ3 taking the most frequent *k*-mers is equally good as choosing the *k*-mers with cross-validation. This means that for those proteins the most frequent *k*-mers are truly the most important ones in classification. On the other hand, for example for HSF2, for which the results are better using the cross-validation, the most important *k*-mers are somewhat longer and therefore also less frequent *k*-mers.

**Figure 4:**
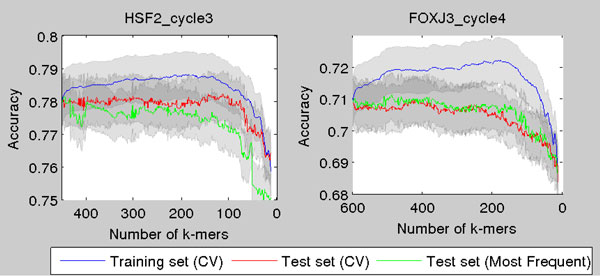
**Illustration of classification accuracy using the CV-scheme when starting with greater number of ***k***-mers**. The test set classification accuracy as a function of number of *k*-mers, when the *k*-mers are chosen with the CV-scheme. The CV is started from 450 and 600 most frequent *k*-mers for proteins HSF2 and FOXJ3. The 95% normal approximation confidence intervals are plotted around the curves.

Cross-validation starting with higher number of *k*-mers yields similar results (Figure 4). Sometimes the most frequent *k*-mers yield equal or even slightly better results than cross-validation scheme, but for some proteins the cross-validation introduces great advantages. For example for HSF2 the cross-validation clearly improves results as the classification accuracy is maximized around 150 *k*-mers and difference between the cross-validated and the enrichment method increases even more for lower smaller number of *k*-mers. Moreover, especially for smaller number of *k*-mers the cross-validated feature selection provides significantly better results than the standard approach (Figures 3 and 4).

**Figure 5:**
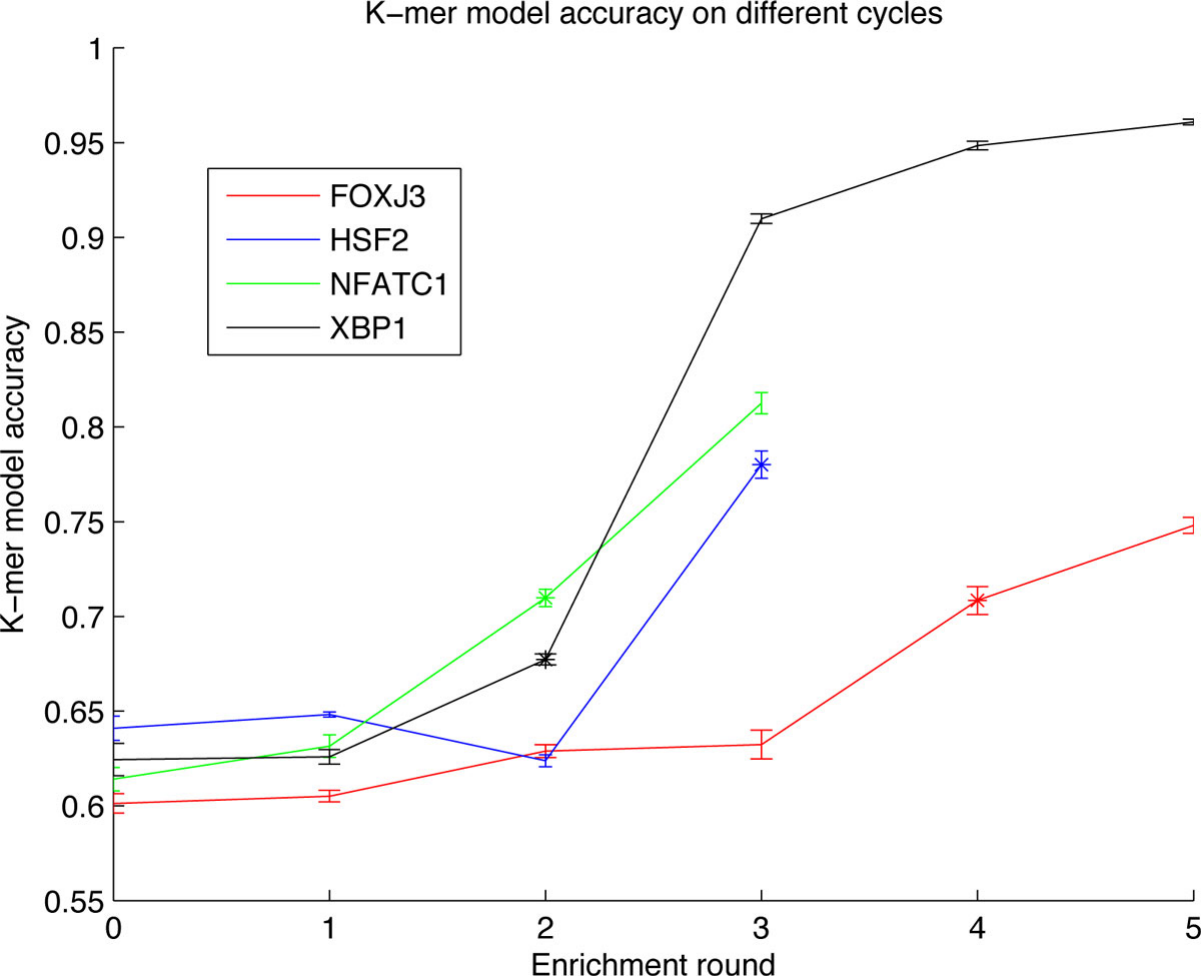
**Classification accuracy in different enrichment cycles**. The classification accuracy from different SELEX enrichment cycles for five proteins. The 95% normal approximation confidence intervals are plotted around the accuracies.

It was also investigated how the chosen SELEX enrichment cycle affects the classification accuracy. The results are plotted in Figure 5. It is clear that the performance is higher at later cycles. This is quite intuitive because the set of sequences should get more homogenous as the enrichment process proceeds. It is also noticeable how classifying the first SELEX rounds is much more difficult than the later cycles. At later cycles there is also much more variability in the results between proteins. The round used in the performance comparison is marked with a star.

**Figure 6:**
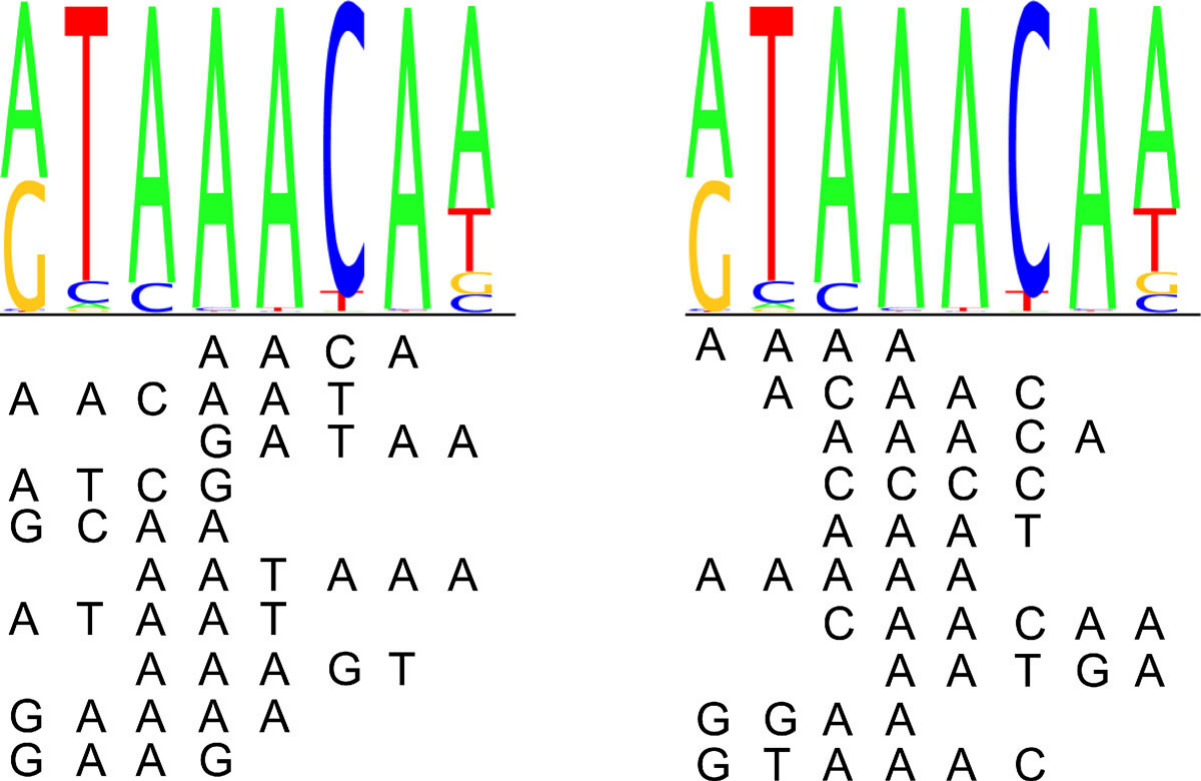
**Top ***k***-mers aligned to FOXJ3 logo**. The top *k*-mers chosen with the CV-scheme (left) and the top affinity *k*-mers from the most frequent approach (right) aligned to FOXJ3 sequence logo.

**Figure 7:**
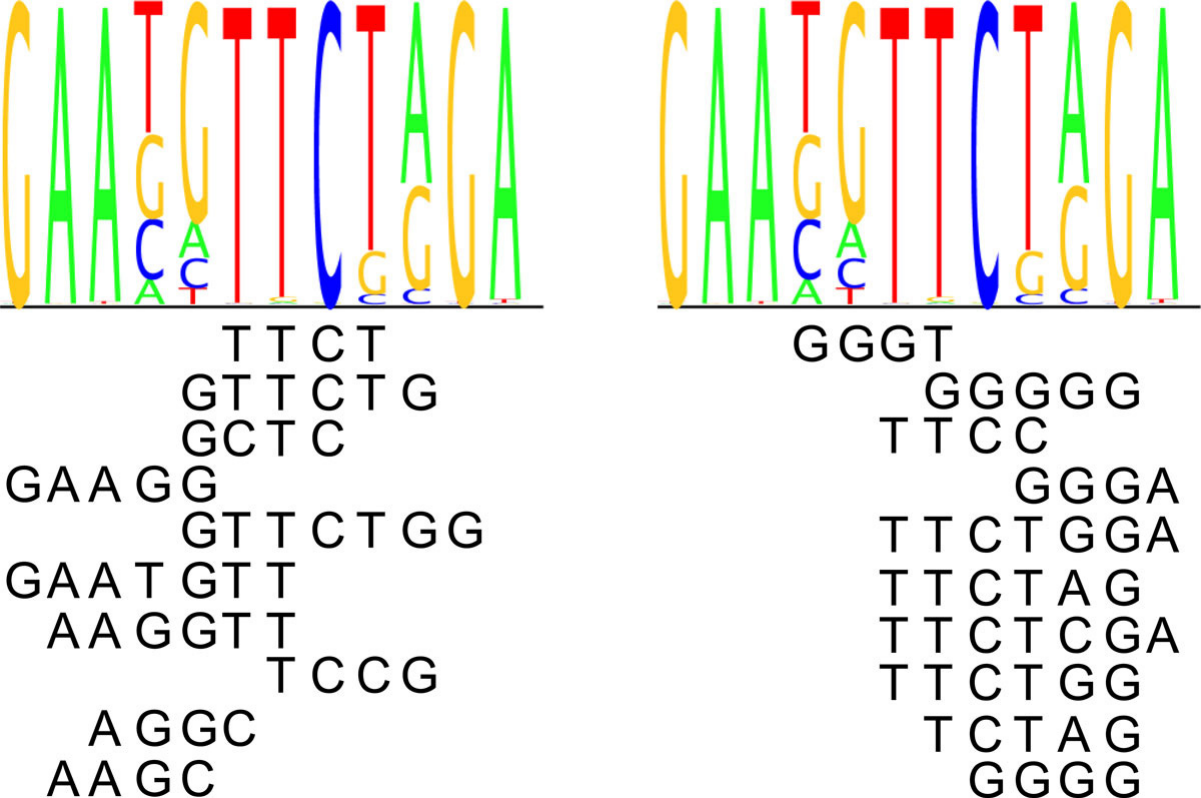
**Top ***k***-mers aligned to HSF2 logo**. The top *k*-mers chosen with the CV-scheme (left) and the top affinity *k*-mers from the most frequent approach (right) aligned to HSF2 sequence logo.

The cross-validation scheme does not choose necessarily those *k*-mers to the model that are important originally with higher number of *k*-mers. It was investigated if the highest affinity *k*-mers would be included late in the cross-validation. There was little overlap between the ten last *k*-mers in the cross-validation and ten most important (highest affinity score) *k*-mers in the most-frequent approach. Nevertheless the *k*-mers are quite similar to each other in the sense that the top *k*-mers in the different methods align relatively well to the PWMs: examples of FOXJ3 and HSF2 are shown in Figures 6 and 7. The *k*-mers aligned to the logo in the left are from the latest rounds in the cross-validation and in the right the *k*-mers are top-affinity *k*-mers from the most-frequent approach. Both *k*-mers and their reverse complements are taken into account when aligning the top *k*-mers to the motifs.

### Application to ChIP-seq data

We also assessed the applicability of the linear *k*-mer model, trained on SELEX data, to ChIP-seq data. That is, the model was still trained with the SELEX data but performance was evaluated using data from ChIP-seq experiments. This required finding suitable data sets for some of the proteins for which SELEX data was produced in [10]. Three data sets, for proteins RFX3, NFATC1 and GATA1, were used. Data for RFX3 and NFATC1 is described in Jolma *et al. *[10] and data for GATA1 was taken from the ENCODE data set. Each data set contained true peaks supposedly bound by the protein of interest, and negative peaks, locations that were chosen randomly and are most likely unbound by the protein.

Area under the curve (AUC) metric was chosen for performance evaluation: the classification threshold previously identified from SELEX data might not be applicable to other types of data, because the reads to be classified are longer. AUC reports the probability that a true bound sequence will score higher than a random non-bound sequence. AUC of 1 corresponds to perfect discovery of the true peaks without making any false positive predictions, and AUC of 0.5 can be reached by classifying the peaks randomly. Mann-Whitney confidence intervals are suitable for estimating the confidence interval of the AUC metric [12]. From each data set AUC was calculated using the entire peak regions that can span several hundred nucleotides and for the centers of each peak (50nt and 100nt regions).

**Figure 8:**
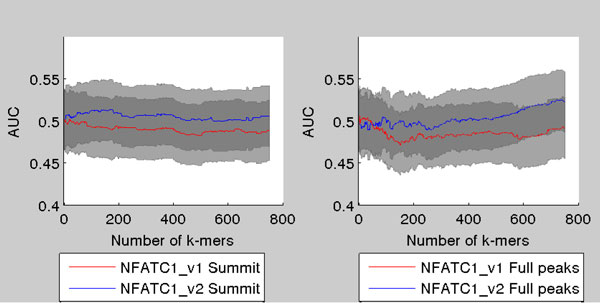
**AUC as a function of number of ***k***-mers in the model for two NFATC1 ChIP-seq samples**. (left) AUCs when the true binding sites are taken to be within 100 nucleotides around the summit of the ChIP-seq peak. (right) The same as (left) except each binding site is taken to be the whole ChIP-seq peak region. The 95% Mann-Whitney confidence intervals plotted around the curves.

For protein NFATC1 AUC is plotted against the number of *k*-mers in the model in Figure 8. As can be seen the AUC stays quite steadily in the range of 0.5 meaning that the *k*-mer model fails to distinquish between true peaks and negative peaks.

**Figure 9:**
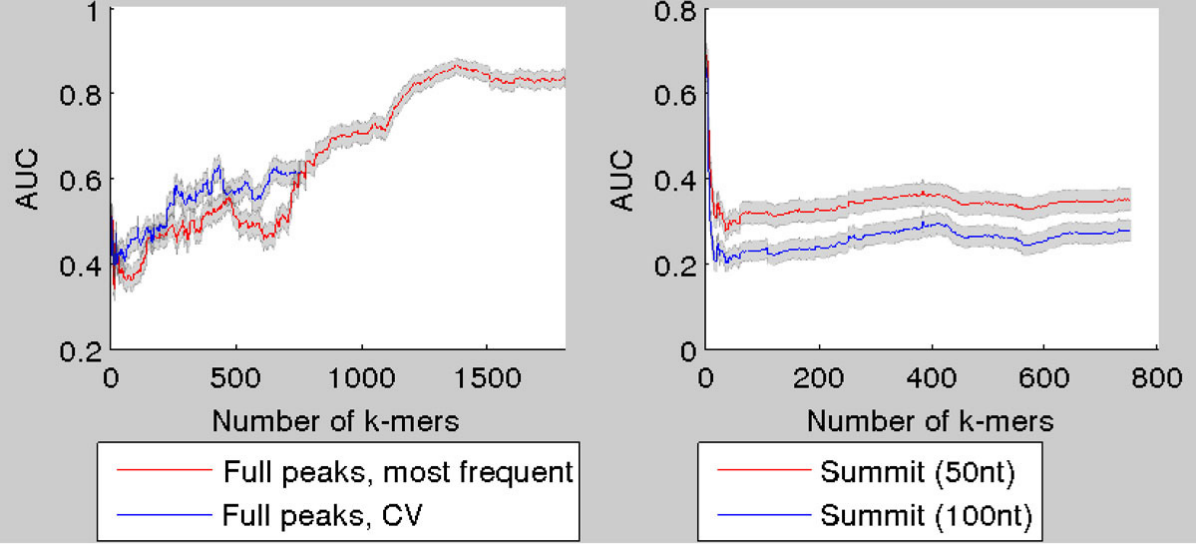
**AUC as a function of number of ***k***-mers in the model for GATA1 ChIP-seq samples**. AUC as a function of number of *k*-mers for GATA1, when *k*-mers are selected with the CV-scheme. In left figure (red) the AUC is plotted when using the most frequent *k*-mers. In right figure the AUC is calculated when only the centers of the ChIP-seq peaks are used. The 95% Mann-Whitney confidence intervals plotted around the curves.

For GATA1 the results are more interesting, as in some cases the *k*-mer model provides great predictive power. The ChIP-seq peaks can be quite accurately distinguished from false peaks as the AUC rises close to 0.9 with relatively high number of *k*-mers in the model (Figure 9, left). The summits in turn can be distinquished from false peaks by using only small number of *k*-mers selected with the cross-validation scheme (Figure 9, right). There is however somewhat controversial phenomenon of AUC dropping significantly below 0.5 when using greater number of *k*-mers in the model. Whether its a property of the data or the *k*-mer model remains to be investigated. Nevertheless, taken together, our results indicate that the previously reported poor performance of the *k*-mer model on *in vivo *data can be improved using careful feature selection strategies.

## Discussion

Feature selection in *k*-mer models can be approached in many different ways. Our results indicate that it is preferable to include the most frequent *k*-mers in the model instead of choosing the most enriched ones. This approach inevitably favours shorter *k*-mers because they are statistically more likely to appear frequently in sequence data. We also observed that, for SELEX data, increasing the number of *k*-mers in the model does not improve predictions after certain point (about 600 *k*-mers). This point most likely changes depending on which kind of data the model is applied to.

The number of *k*-mers can be reduced without great negative effects in prediction accuracy using the standard cross-validation scheme. Consequently, this decreases the number of parameters which need to be estimated for the *k*-mer models making them more attractive and applicable binding prediction model.

Although it is quite clear that the *k*-mer model outperforms widely used PWM-models within the SELEX data set, the performance of the *k*-mer model with *in vivo *data still remains an open question. For two data sets (proteins NFATC1 and RFX3) the *k*-mer model failed to distinquish between true binding sites and unbound sites. For GATA1 the results however seem very promising as the AUC of close to 0.9 was reached.

## Competing interests

The authors declare that they have no competing interests.

## Authors' contributions

JK analyzed the data, collected results and wrote the paper. HL designed and supervised the study and wrote the paper.

## Declarations

The publication costs for this article are covered by Academy of Finland Center of Excellence in Systems Immunology and Physiology.

This article has been published as part of BMC Bioinformatics Volume 14 Supplement 10, 2013: Selected articles from the 10th International Workshop on Computational Systems Biology (WCSB) 2013: Bioinformatics. The full contents of the supplement are available online at http://www.biomedcentral.com/bmcbioinformatics/supplements/14/S10
